# Smart ECG Biosensor Design with an Improved ANN Performance Based on the Taguchi Optimizer

**DOI:** 10.3390/bioengineering9090482

**Published:** 2022-09-19

**Authors:** Lilia Sidhom, Ines Chihi, Mahfoudh Barhoumi, Nesrine Ben Afia, Ernest Nlandu Kamavuako, Mohamed Trabelsi

**Affiliations:** 1 National Engineering School of Bizerta, Carthage University, Tunis 7035, Tunisia; 2 Laboratory of Energy Applications and Renewable Energy Efficiency (LAPER), Faculty of Sciences of Tunis, El Manar University, Tunis 1068, Tunisia; 3 Faculty of Science, Technology and Medicine, Department of Engineering, University of Luxembourg, Kirchberg, L-135 Luxembourg, Luxembourg; 4 National Engineering School of Monastir, University of Monastir, Monastir 5019, Tunisia; 5 Department of Engineering, King’s College London, London WC2R 2LS, UK; 6 Electronics and Communications Engineering Department, Kuwait College of Science and Technology, Kuwait City 1613, Kuwait

**Keywords:** ECG prediction, measurement sites, Artificial Neural Network ANN, taguchi method, optimization, real database

## Abstract

This paper aims to design a smart biosensor to predict electrocardiogram (ECG) signals in a specific auscultation site from other ECG signals measured from other measurement sites. The proposed design is based on a hybrid architecture using the Artificial Neural Networks (ANNs) model and Taguchi optimizer to avoid the ANN issues related to hyperparameters and to improve its accuracy. The proposed approach aims to optimize the number and type of inputs to be considered for the ANN model. Indeed, different combinations are considered in order to find the optimal input combination for the best prediction quality. By identifying the factors that influence a model’s prediction and their degree of importance via the modified Taguchi optimizer, the developed biosensor improves the prediction accuracy of ECG signals collected from different auscultation sites compared to the ANN-based biosensor. Based on an actual database, the simulation results show that this improvement is significant; it can reach more than 94% accuracy.

## 1. Introduction

Nowadays, body sensors monitor heart signals with a high precision and negligible noise. Smart sensors are considered an alternative solution to physical sensors. In heart disease applications, smart sensors are used to predict a biomedical signal, such as the ECG, based on the history of that signal or to predict a signal using other available means. 

ECG electrodes are sensitive to several distortions and may be damaged by certain environmental factors. The displacement of electrodes from the desired measurement site or the detachment of the transmission cable could change the temporal and frequential characteristics of ECG signals, which may lead to a false analysis and poor measurement quality. Innovative biosensors are considered an alternative solution to reduce noise and signal cuts in case of external or internal perturbation. The intelligent sensors are also used to reduce the number of electrodes placed on the patient’s chest to ensure a proper interpretation and diagnosis of the heart health condition. 

Smart sensors are generally based on machine learning (ML) methods. Among the most popular and widely used ML techniques is the ANN. Indeed, this technique has been successfully used in various application areas such as climate [1], energy [2], and communication technology [3]. The literature presents different techniques of ECG biosensors. In [4], the authors proposed an ECG biosensor based on the autoregressive integrated moving average (ARIMA) model with a discrete wavelet transform. Despite the good prediction of the amplitude, it should be noted that the assumption of a local linearity limits such a method. To avoid this drawback, other ECG biosensors based on the ANN model are also used to predict ECG signals [5,6]. In [5], the authors applied the ANN to detect the ECG abnormalities by using a multilayer perceptron network trained by three different algorithms such as the Bayesian regularization (BR), the Levenberg–Marquardt (LM) and backpropagation (BP). The obtained results are satisfactory. Other biosensors, such as [7,8] can also be cited. In [7], Gutta et al. proposed a biosensor allowing the extraction features and classification of ECG signals using a recurrent neural network. [8] proposed another ECG identification algorithm based on the support vector machine.

Despite the multitude of existing ECG biosensors, the one based on the ANN is of great interest. However, despite its success in various applications, ANN methods have different limitations. The two most known issues that have a substantial impact on the estimation quality are catastrophic forgetting (CF) and the choice of hyperparameters [9,10]. Indeed, CF is the loss of information related to a first task after training for a second task. This information declination could almost be catastrophic in some cases [11,12,13,14,15]. For example, the problem of using many hidden layers with a nonlinear activation function is that the proposed ANN model can stop learning, leading to poor results. Indeed, the so-called vanishing gradient problem is highlighted in this case. It is caused by the information expression that takes place at each iteration. From a practical standpoint, rectified linear units (ReLU) present an excellent function. Many models also rely heavily on ReLU to give good results since it preserves the information. However, this function has its disadvantages; for example, it runs into a dying ReLU problem in rare cases and can block the negative values that prevent the propagation of certain information. 

Hyperparameter optimization is another issue that can affect the ANN prediction efficiency. In this perspective, many researchers suggested using the random search method [16,17]. This technique consists of generating architectures with different parameter values. The grid search method also relies on checking all possible architectures with a limited set of parameters defined values [18]. However, this will require a lot of resources and can be inefficient and time-consuming, even impossible when the problem presents a high complexity. The output is a nonlinear combination of the input variables. Indeed, the ANN method finds the best neural network architecture and determines the weight using retro-propagation. The retro-propagation algorithm is applied at each iteration to update the weights. Decreasing the number of epochs will thus not provide good results but increase the number of epochs which might lead to over-fitting. Therefore, it is advised to try different combinations to determine the adequate values.

To solve the ANN hyperparameter problem, several optimization techniques were proposed in the literature. Conventional methods such as gradient descent [19] and graph theory [20] have some constraints mainly defined by the local optimization and unknown search space problems. Moreover, heuristic and metaheuristic methods have been used. These techniques are divided into four subclasses: Algorithms based on swarm intelligence such as the Harris Hawks optimization (HHO) [21] and the genetic algorithm (GA) [22], evolutionary algorithms, natural phenomena-based algorithms, and natural sciences-based algorithms such as the Henry gas solubility optimization (HGSO) [23]. In the latter, the authors adapted the HGSO algorithm (based on Henry’s law for the imitation of gas behavior) to tune the random forest’s hyperparameters in the prediction of caesarean births. In [24], the authors proposed an improved prediction algorithm based on the HHO method with the aim of assessing early the severity of COVID-19. Similarly, the authors in [25] proposed an optimized machine learning approach by combining the Harris Hawk method with the feature analysis based on the SHapely adaptive exPlanations (SHAP) method for a better COVID-19 prediction. Over the last decade, nature-inspired algorithms and swarm intelligence have been increasingly used and applied in different disciplines due to their efficiencies and flexibility. The major advantages they have are black-box and gradient-free optimizers. However, there are some key issues regarding these algorithms concerning their analysis in terms of their stability, convergence, convergence rate, and robustness. In [26], an in-depth review of some recent nature-inspired algorithms emphasizes five open issues regarding the analysis of algorithmic convergence, parameter tuning, role of calibration, etc. 

From this perspective, the main idea of this paper is to propose a smart ECG biosensor defined by a hybrid architecture based on ANNs and the Taguchi optimizer. This optimizer is a powerful and efficient statistical tool for the design of high-quality systems [27,28]. It is used for different kinds of applications [29,30,31,32,33]. The Taguchi has also proved its effectiveness as a robust tool for deep learning applications [34,35]. It is well-known for its robustness-based orthogonal array design. The Taguchi optimizer allows reducing the time needed to study the influence of individual factors and determine which factor has more influence by applying the simplest technique with any mathematical complexity. According to [36], a comparative study between the Taguchi method and some genetic algorithms (GA) was performed. It is proven that it is preferred to use the Taguchi method rather than GA, given the cost of the experiment design, the number of experiments, quality, and performance. Such an approach consists of narrowing the search field after each iteration by checking the statistics of the result on each iteration and readjusting the search parameters to predict the measured ECG signals in different locations. In summary, this optimizer reduces the computational time, avoiding the problems associated with the hyperparameters and the computing time related to the ANN predator.

The contributions of this work are multifold. The Taguchi optimizer layer has been applied on ECG signal prediction for the first time with a high accuracy, enabling the optimization of the number of body ECG sensors. It is well known that the most common method to reconstruct the 12-lead ECG from a limited lead set is a linear regression. Despite the use of ANNs in the literature [37], the enhancement of ANNs through the Taguchi optimizer has never been attempted before. While many papers [38,39] have proposed an ECG signal reconstruction of missing samples, their methods are mainly based on multiple ECG leads that are linearly connected. To the best of our knowledge, no other paper has attempted the prediction of ECG using independent traces. This novel approach is significant and challenging as the reconstruction is based on single leads measured at different auscultation sites. The clinical applications of the proposed approach include designing and implementing miniaturized devices for measuring and tracking heart diseases. Such devices are suggested to make use of ECGs to drive the segmentation of phonocardiograms [39]. Furthermore, the field of monitoring and tracking utilizes telemedicine to transmit data through various connectionless channels. These channels are often affected by the loss of data packages (occurring during transmission) that need to be recovered. While reconstructed from multiple leads, the ECG has received much attention [40], and novel methods are required for independent channels. The importance of data reconstruction has proven to be very relevant in the remote monitoring of patients in rural areas [41]. 

This paper is structured as follows. Section 2 introduces the ECG biosensor design with an overview on the prediction approach. Then, the Taguchi-based ANN model is discussed in detail. For that, the basics of this method are recalled and applied to a study case. The simulation results with a comprehensive performance comparison between the basic ANN algorithm and the proposed Taguchi-based ANN are presented in Section 3. Last, Section 4 concludes the paper. 

## 2. ECG Biosensor Design

**Figure 1:**
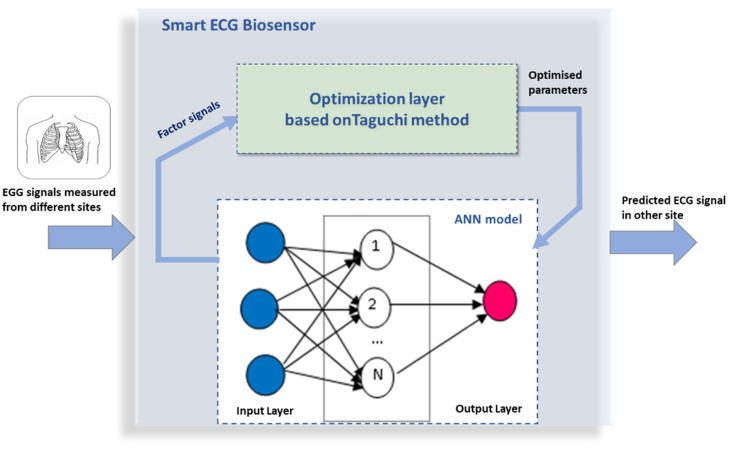
Overview of the architecture of the proposed ECG biosensor.

The architecture’s overview of the proposed ECG biosensor is described in Figure 1. The proposed design includes two layers. The first one is presented by the Taguchi-based optimization layer, while the ANN model defines the second layer. As already mentioned, the Taguchi optimizer mitigates the ANN model’s hyperparameters (loss of function, number of layers, number of epochs, optimizer type, etc.) optimization issue. The biosensor design computes one output which is defined by the predicted ECG signal.

The input number of the ANN model is variable to the predicted ECG signal. In fact, the number and type of ANN inputs are defined by different combinations of ECG signals measured at other sites. With the Taguchi method, these combinations are considered to have the best prediction quality. Prior to starting the different steps of the optimization Taguchi procedure, it is crucial to understand all of the factors influencing the ANN performance. Based on the literature review, it is easy to detect these factors and identify them as input signals to the optimization layer based on the Taguchi method. These signals are noted signal factors, see Figure 1. Then, the different optimization steps will be explained in the following to obtain the optimized hyperparameters with which the response of the ANN model will be enhanced.

### 2.1. General Information on the Prediction Procedure 

**Figure 2:**
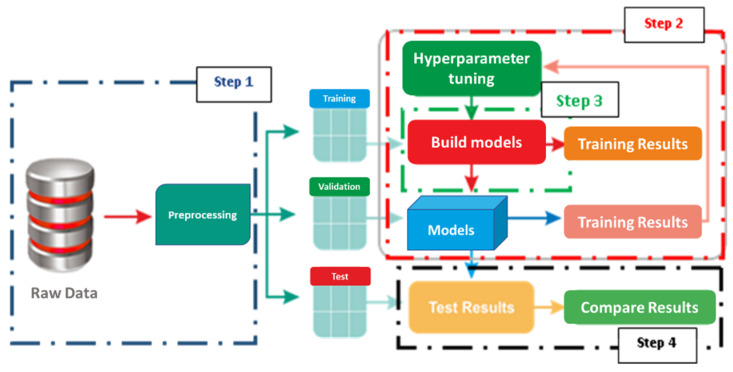
Block diagram of the proposed prediction of ECG signals.

Figure 2 shows the four steps of the proposed design. In the first step (pre-processing), the data is divided into training and testing sets. Step 2 consists of building models based on the Taguchi optimizer by defining the interval of values for each parameter. The model structure is then defined in Step 3 by mentioning the hyperparameters, which will be tweaked through trial and error, and their possible values. For each trial/hyperparameter configuration, the weights of the best epochs are saved along with the metrics evaluation and then processed. Finally, Step 4 is dedicated to visualizing and analyzing the efficiency of the predicted ECG output. This step compares the results obtained with/without the use of the Taguchi optimizer. 

The data collection was performed on 10 healthy subjects in London. The database is acquired at King’s College London, London WC2R 2LS, UK [41,42]. The King’s research Ethics Committee approved the experimentation (Approval No.: LRS-18/19-10673). Table 1 summarizes some properties related to the data acquisition procedure that can influence the data quality.

**Figure 3:**
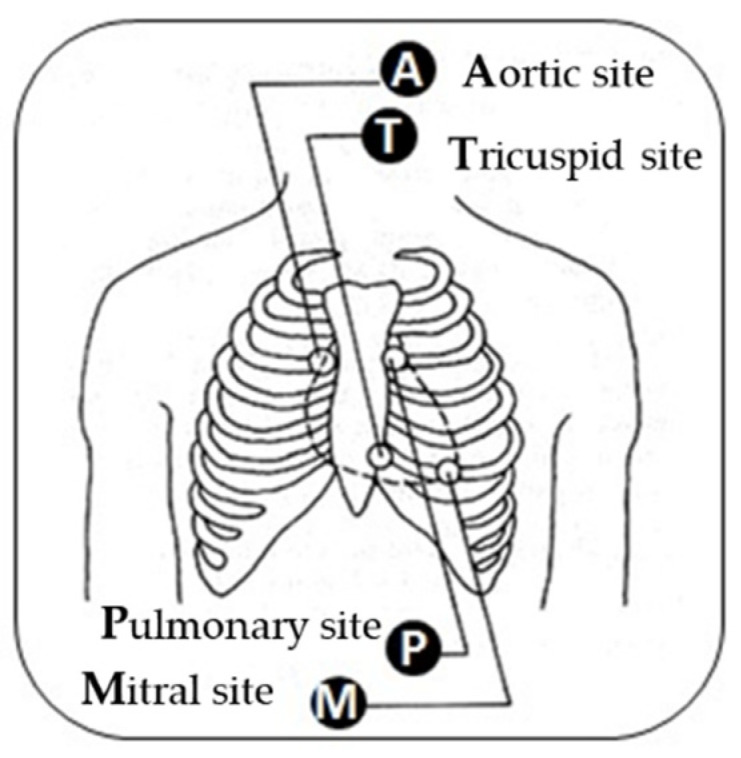
The auscultation sites. (**A**) **A**ortic site (ECG**A**): on the patient’s right side of the sternum. (**P**) **P**ulmonary site (ECG**P**): on the left-hand side of the patient’s sternum. (**T**) **T**ricuspid site (ECG**T**): in the fourth intercostals space, along the lower-left border of the sternum. (**M**) **M**itral site (ECG**M**): along the mid-clavicular line in the fifth intercostal space.

ECG signals were collected on Lead I and the four auscultation sites were described in [41,43]. During the recording, only three signals were collected at a time: Lead I, ECGA, and a signal from the other three sites as it is illustrated in Figure 3. Since signals are time-dependent, each set of signals will be treated separately.

Filtering the ECG data is a crucial step since the data contains noise which is due to many external/internal factors (respiration, vibration, sensitivity of the sensors, etc.). In this case study, a third-order Butterworth filter is used. An automated adaptation algorithm that gives the best signal-to-noise ratio is performed to set the correct filter cut-off frequency for each subject to find the best setting filter. Based on the min-max method [44], the data is normalized, and the signals’ magnitude is defined between 0 and 1. The normalization step is important to improve the estimation performance by decreasing the sensitivity of the weight values and making it easy to adjust. 

Following the cleaning and filtering the data, the statistical features of the different measured ECG signals were computed by Table 2.

Data normalization and correlation study are then proposed. Table 3 presents the correlation study of the used data. It shows low, medium, and high correlation coefficients, which means that the linear models will not be adequate for the low correlated sets. In addition, in case of many measurement points, errors will sum up which leads to the deteriorated results.

### 2.2. Taguchi Optimizer-Based ANN Model

#### 2.2.1. Background of the Taguchi Method

The Taguchi method offers powerful optimization achievements for products or processes. It makes possible the parameter design to reduce the possible variation, which makes them robust and flexible. Further details about the Taguchi method can be found in [33,44,45]. 

**Figure 4:**
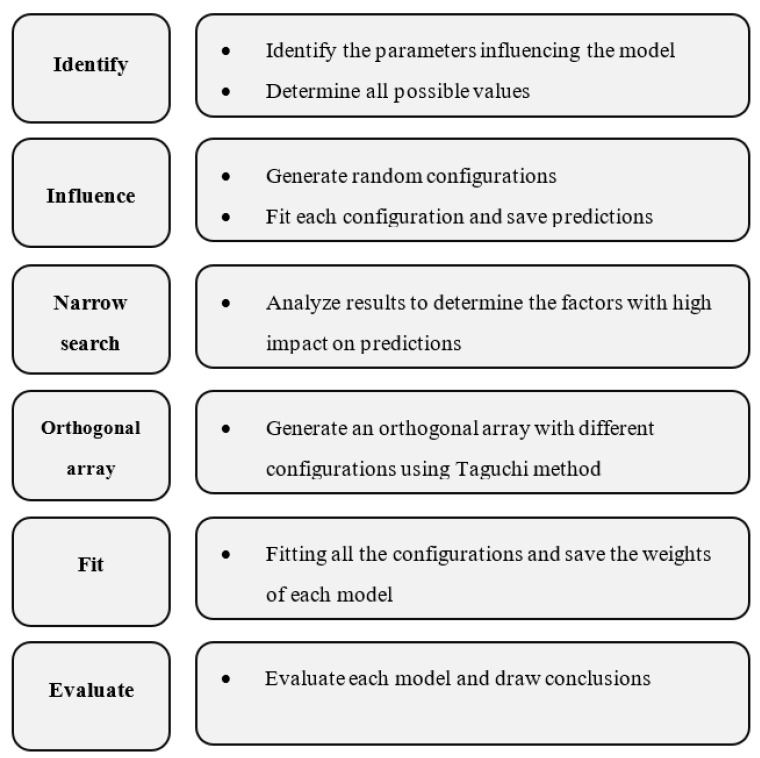
Diagram of the Taguchi method.

As presented in Figure 4, the application of this method in ML can be illustrated in six steps. The first three steps limit the search field by identifying the factor signals and their effects on the fitting process [45,46]. This can be carried out by determining the parameters needed to design the ANN architecture. Then, to check the influence of each parameter, an architecture configuration should be selected while keep changing only a specific one to study its impact on the results. If it has no influence, its value will be unchanged while searching for the best parameters, to save resources such as time and memory. Therefore, the idea consists of making an orthogonal array that contains different configurations of these factors. 

Following the fitting, each architecture will be evaluated based on the objective functions/metrics. These metrics help to determine which parameters enhance the model’s findings, thus narrowing the search field. For our case, the chosen performance indices are defined by Equations (1)–(3). 

Coefficient of Determination (*R*^2^):

(1) $$R^{2}=\sqrt{\frac{\sum_{i=1}^{N}{\left(Y_{i}-\bar{Y_{i}}\right)}^{2}-\sum_{i=1}^{N}{\left(Y_{i}-\hat{Y_{i}}\right)}^{2}}{\sum_{i=1}^{N}{\left(Y_{i}-\bar{Y_{i}}\right)}^{2}}}\;$$

This coefficient represents the variability measure of the reproduced data in the model.

Mean absolute error (*MAE*):

(2) $$MAE=\frac{1}{n}\overset{N}{\underset{i=1}{\sum }}\left|\left(\hat{Y_{i}}-Y_{i}\right)\right|$$

Mean squared error (*MSE*):

(3) $$MSE=\frac{1}{n}\overset{N}{\underset{i=1}{\sum }}{\left(Y_{i}-\hat{Y_{i}}\right)}^{2}$$

Standard deviation (*SD*):

(4) $$SD=\sqrt{\frac{\sum_{i=1}^{N}\left(Y_{i}-\bar{Y}\right)}{N-1}}$$

The *MAE* and *MSE* parameters provide a general idea of the difference between the modelled and the observed values. 

*MAE*, *MSE* and *R*^2^ are used to select the best neural network [46].

where:

*N* = number of data points
$Y_{i}\;$= observed values
$\bar{Y}$ = mean values
$\hat{Y_{i}}$ = predicted values

Since we are dealing with a regression problem, we should use dense layers. For the activation function [45], the most common were *Relu*, *Elu*, *Selu*, *Sigmoid*, *Tanh* and *Linear*, which are defined as follows:

(5) $$\mathrm{Relu}:\;f\left(x\right)=\{\begin{array}{c} x\;\;\;\;\;\;\;\;\;if\;x>0\\ 0\;\;\;\;\;Otherwise \end{array}$$

(6) $$\mathrm{Elu}:\;f\left(x\right)=\{\begin{array}{c} x\;\;\;\;\;\;\;\;\;if\;x>0\\ \alpha .\left(e^{x}-1\right)\;\;\;\;\;Otherwise \end{array}\;\;\;\;\;\;\;\;\;\;\;\alpha =1\;\;\;\;\;\;\;\;\;\;\;\;\;$$

(7) $$\mathrm{Selu}:\;f\left(x\right)=\{\begin{array}{c} x\;\;\;\;\;\;if\;x>0\\ \alpha .\beta .\left(e^{x}-1\right)\;\;Otherwise \end{array}\;\;\;\alpha =1.76\;,\beta =1.05$$

(8) $$\mathrm{Sigmoid}:\;f\left(x\right)=\frac{1}{1+e^{-x}}$$

(9) $$\mathrm{Selu}:\;f\left(x\right)=\frac{e^{x}-e^{-x}}{e^{x}+e^{-x}}$$

(10) Linear:   f(x)=x  

The number of hidden layers varies between three and eight, with each layer having a unit not exceeding 255. As for the objective function, it is either (mean absolute error) *MAE* or (mean squared error) *MSE*, respectively, and defined by Equations (2) and (3). Another objective function, *VAL_LOSS*, is also used, which is determined by the value of the cost function for cross-validation, meaning the mean squared error calculated while running tests on the dataset test. 

#### 2.2.2. Taguchi Method Implementation with ANNs 

The first step in implementing the Taguchi method is to identify the neural network parameters (or factor signals) that will be modified. Since the number of neurons in the input and output layers of the ANN is constant, the number of hidden layers, activation functions, number of epochs, objective function, and optimizers to train-test the split rate are unknown.

**Figure 5:**
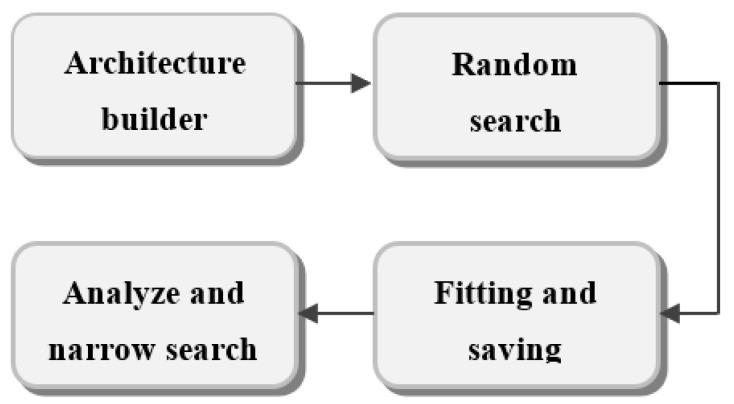
Diagram of the parameter selection process.

Unlike other conventional experimental designs, the particularities of the Taguchi method are illustrated in Figure 5. This method allows the identification of the principal factors responsible for the problem and their importance to the optimal solution [47]. 

**Figure 6:**
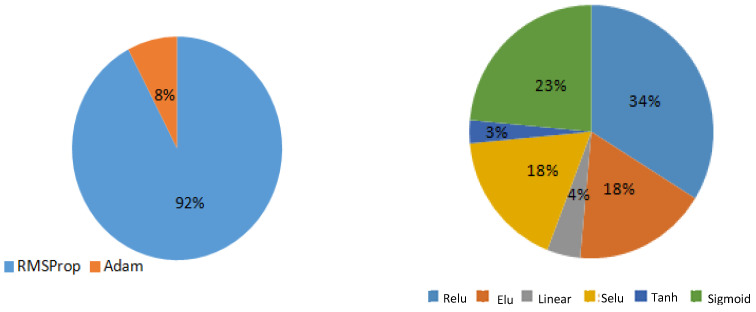
Statistics of the parameters of the best results.

Using Keras Tuner [48], a library defined in Keras, facilitates the tuning of the neural network by automating the operation of parameter changing. This operation is essential to determine the interval or choices for each parameter in the architecture builder. It is then necessary to generate different possible configurations. Increasing the number of possibilities will take a lot of time for the machine to search for the best weights for each combination. In addition, narrowing the choices hypothetically can induce poor results; thus, a primary random search was conducted by fixing the number of trials. A trial is running a search with a predefined configuration. This method helps to narrow the search field since each configuration generated by the architecture builder is fitted after every set of trials. Following this, the results are analyzed, and the parameter values that obtained the best predictions are determined. A primary random search with a wide range of values for each parameter has been conducted; the number of trials was fixed at 200. At the end of the first search, the results were analyzed to determine the impact of each parameter. The trials with the best results were analyzed to narrow the parameters summarized in Figure 6. From this figure, the statistics have shown that the linear and tanh(.) activation functions were almost absent from the architecture of the best results. In addition, the sigmoid function was, for most of the cases, present in the final hidden layer [49]. The number of hidden layers varies from three to six, with the same proportions for each. As for the optimizer, SGD [50], a gradient-based optimization technique along with RMSProp [51] and Adam [52], was absent from the top ten results, and Adam was only present once; thus, we opt for the use of RMSProp in the future. Other parameters, such as the loss function train-test ratio and the number of epochs, were diverse in their results; thus, when designing the Taguchi table of parameters, we opt for spreading the values of these parameters. A checkpoint callback function was added to preserve the weights of the optimal model, and the train-test ratio was fixed at 80–20. 

The results given in Figure 6 are then summarized in Table 4 to present the ANN parameters for the random search without optimization. The best ECG forecasting results with the optimized ANN model are shown in Table 5. 

In the next step, an orthogonal array was designed based on the freedom degrees of each parameter, as it is detailed by Taguchi [53]. 

Coming to this level, the search field is clear; the next phase is running the different combinations and evaluating the results, which will be detailed in Section 4.

## 3. Results and Discussion

This section tackles the numerical validation of the ECG biosensor based on the performance of the optimized ANN model using the technical and software requirements described in Table 6.

With five signals available, one signal will be the output while the remaining signals are combined to form the input data. Due to the data collection method and in order to reduce the search field, it is advisable to lower the amount of content with three signals, Lead1, ECGA, and either ECGP or ECGT or ECGM, narrowing the possibilities to only 27.

**Figure 7:**
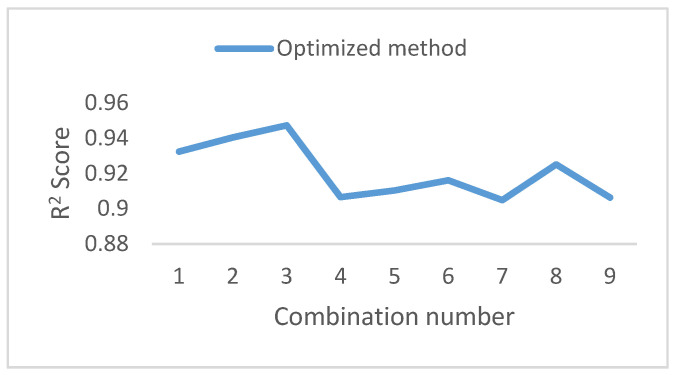
*R*^2^ Values for the different number of inputs: Optimized method results.

**Figure 8:**
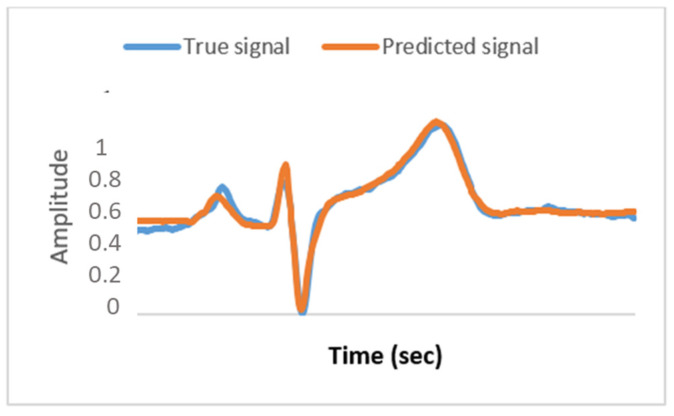
Response of the proposed method: ECG prediction plot.

Figure 7 shows a high *R^2^* score for all of the possible combinations of ECG signals measured in different sites; for example, ECGP, ECGA, and LEAD1, using LEAD1 as an input and ECGA as an output. As for the signals ECGT and ECGM, the R2 score was between 50% and 80%, which is due to the low correlation factor, unlike the case with ECGP, which makes this site a good location to rely on for predicting other signals demonstrated in Figure 7. We note that the best efficiency estimation is shown in Figure 8. It is the result of the use of ECGP and ECGA to predict LEAD1 of subject nine and of a neural network’s architecture based on seven hidden layers 100–100–10–10–200–10–200, respectively, with activation functions: Selu-Selu-Relu-Relu-Elu-Selu-Sigmoid.

**Figure 9:**
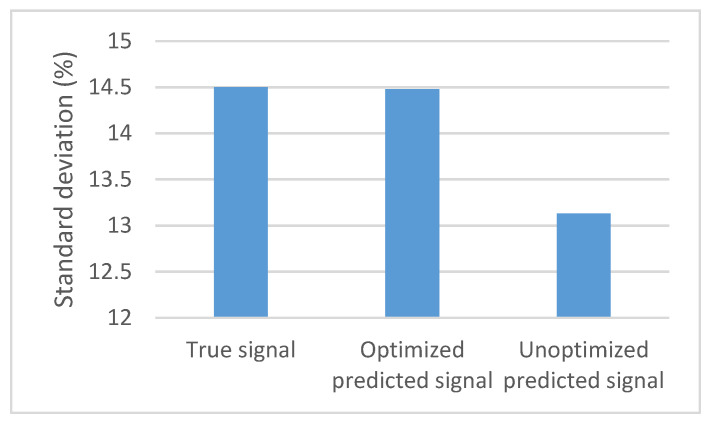
Data distribution of the different methods.

The predicted signal’s standard deviation (SD) with the optimized method rounds up to 0.1448. The actual signal’s SD rounds up to 0.1450, whereas the signal obtained from the random search only method results in an SD of 0.1313, which is far from the actual value. As shown in Figure 9, both the actual signal and the predicted signals using the Taguchi method show many similarities in the median values with a 0.57% error, upper quartile, lower quartile, maximum, and minimum values with a 1.3% error between the interval of points. 

The signal predicted by the random search only presents a 4.7% error in the median value and an 18% error in the maximum and minimum values compared to the actual signal. Consequently, the properties of the signal are entirely changed. ECG signals are very sensitive and delicate, and such errors can change their characteristics.

Via the proposed prediction approach, it is also possible to know how many model inputs and which ECG signal sites to use to obtain the best result. Indeed, the prediction with an optimized search proves that estimating an ECG signal via two ECG inputs instead of one is more accurate with respect to the imposed performance criteria.

**Figure 10:**
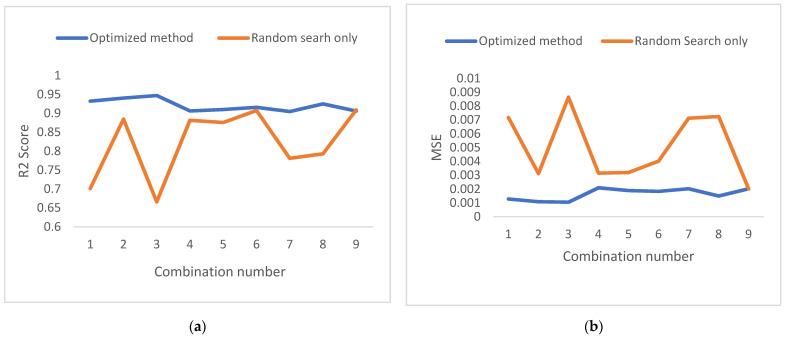

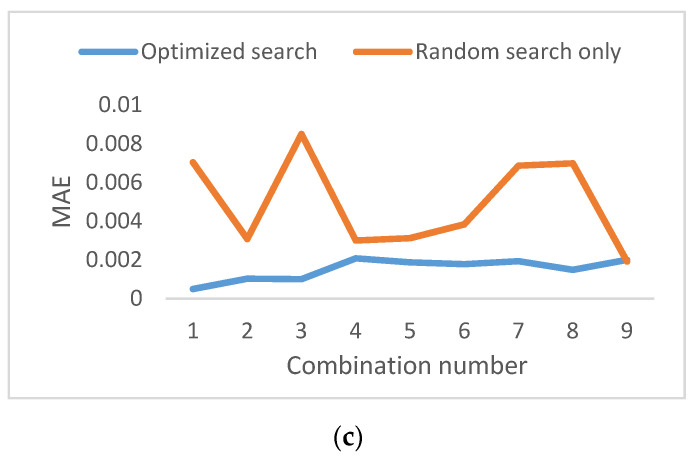
Comparison of the Taguchi method with the random search method for the signals ECGA, ECGP, and LEAD1. (**a**) *R*^2^ Values, (**b**) MSE Values, (**c**) MAE Values.

To verify the efficiency of the Taguchi method, we reran the search randomly using the random search function on the Keras tuner, and the difference was noticeable in Figure 10.

The percentage of the trials in the search field is less than 0.1%, which means that the number of tests for each combination is minimal. That is why the random search method gives poor results. Running all of the input combinations for the random search method took more than 15 h for each type of signal (ECGP, ECGT, or ECGM), whereas in the optimized method, it took almost six hours and gave better and more consistent results.

As shown in Figure 10a, the searches with an *R*^2^ score of 94% were the best results, with ECGP or Lead1 as an output signal. This method was accurate because of the small sample taken, and ECGP and Lead1 have a high correlation factor with the other signals.

In conclusion, the ANN model optimized with the Taguchi method gives better results than those obtained via a random search. In addition, if the data presents a medium-low correlation coefficient, this method is preferred since it cuts a lot of research time and eliminates unnecessary parameter values.

For the same database, a comparative study between different estimators (Linear Regression (LR), K-nearest neighbors (KNN), random forest regression (RFR) and ANNs) was proposed in [54] to predict the ECG signals measured in different sites. It was shown that the RFR and KNN models perform better in terms of prediction efficiency than the LR model in direct and cross validation.

Table 7 summarizes the MSE, MAE, and *R*^2^ values that represent the average values obtained for all of subjects for the proposed optimized approach and for the LR, KNN, RFR, and ANN algorithms. These results show the interest of the proposed estimator compared with the other.

## 4. Conclusions

This paper proposed a new design for a smart ANN-based ECG biosensor using the Taguchi method. It has been shown that the inclusion of the optimization layer in the biosensor architecture improved the ANN performances by saving the used resources and enhancing the prediction quality. To evaluate the effectiveness of the proposed design, a comparative study was carried out between the classical ANN model and the optimized one. Various performance metrics were exploited to ascertain the effectiveness of the proposed ECG biosensor. It can be concluded that the optimized scheme can more reliably predict the ECG signals than the basic one. Moreover, the execution time was reduced more than a half.

Furthermore, it is worth noting that the number and the type of the ANN inputs were also considered in the parameters’ optimization. The results obtained here are a step forward in the application of an independent ECG trace reconstruction in the telemetry monitoring of patients and the design of miniaturized combined Electrocardiogram-Phonocardiogram devices. 

## Figures and Tables

**Table 1:** Important properties of the data acquisition procedure.

Property	Description
Respiratory or heart diseases	No
Data acquisition frequency	20 kHz
ECG sensor	Solid gel electrodes, Ambu WS, size: 36 × 40 mm, Medico Electrodes International LTD., Uttar Pradesh, India
Recording device	iWire-BIO4, iWorx Systems Inc., Dover, NH, USA
Analog filter	0.05–40 Hz
Number of samples	3,628,032
Size of the database	249.1 MB

**Table 2:** Statistical features of 10 subjects.

		Mean	Median	STD			Mean	Median	STD
**Sub_1**	**ECGT**	0.31	0.21	0.24	**Sub_6**	**ECGT**	0.4	0.38	0.17
	**ECGP**	0.41	0.33	0.23		**ECGP**	0.55	0.57	0.17
	**ECGM**	0.4	0.29	0.22		**ECGM**	0.45	0.38	0.25
	**ECGA**	0.36	0.35	0.1		**ECGA**	0.25	0.24	0.12
**Sub_2**	**ECGT**	0.58	0.6	0.15	**Sub_7**	**ECGT**	0.53	0.46	0.21
	**ECGP**	0.54	0.54	0.12		**ECGP**	0.33	0.23	0.22
	**ECGM**	0.36	0.34	0.12		**ECGM**	0.33	0.3	0.15
	**ECGA**	0.32	0.3	0.11		**ECGA**	0.38	0.39	0.14
**Sub_3**	**ECGT**	0.62	0.66	0.15	**Sub_8**	**ECGT**	0.55	0.54	0.15
	**ECGP**	0.28	0.29	0.2		**ECGP**	0.49	0.43	0.2
	**ECGM**	0.31	0.34	0.16		**ECGM**	0.59	0.59	0.12
	**ECGA**	0.59	0.71	0.2		**ECGA**	0.37	0.39	0.15
**Sub_4**	**ECGT**	0.67	0.73	0.19	**Sub_9**	**ECGT**	0.52	0.55	0.17
	**ECGP**	0.42	0.43	0.12		**ECGP**	0.61	0.62	0.13
	**ECGM**	0.64	0.7	0.2		**ECGM**	0.52	0.51	0.11
	**ECGA**	0.51	0.54	0.14		**ECGA**	0.29	0.3	0.1
**Sub_5**	**ECGT**	0.47	0.42	0.14	**Sub_10**	**ECGT**	0.4	0.38	0.17
	**ECGP**	0.47	0.47	0.11		**ECGP**	0.55	0.57	0.17
	**ECGM**	0.42	0.34	0.17		**ECGM**	0.45	0.38	0.25
	**ECGA**	0.36	0.35	0.14		**ECGA**	0.5	0.52	0.18

**Table 3:** Rate correlation in (%) between ECG signals measured in different auscultation sites.

	ECGA	ECGM	LEAD I
ECGA	100%	32.1%	52%
ECGM	32.1%	100%	25.7%
LEAD I	52%	25.7%	100%
	ECGA	ECGT	LEAD I
ECGA	100%	51.8%	60.3%
ECGT	51.8%	100%	42.9%
LEAD I	60.3%	42.9%	100%
	ECGA	ECGP	LEAD I
ECGA	100%	39.1%	30.6%
ECGP	39.1%	100%	38.4%
LEAD I	30.6%	38.4%	100%

**Table 4:** Taguchi table of parameters assigned to their possible values (Input parameters for the non-optimized ANN)**.**

Parameter	Type/Value
Activation Function	Relu (4)-Elu(5)-Selu(6)- Sigmoid (7)
Number of hidden layers	3–4–5–6
Number of units in each layer	Between 5 and 255
Optimizer	RMSProp
Loss function	MSE-MAE-VAL_LOSS
Number of Epochs of each trial	10–100–200
Callback functions	Early stopping (10 patience)-Checkpoint
Train-Test Ratio	0.8–0.2

**Table 5:** Input parameters for the best results obtained with the proposed ECG biosensor.

Parameter	Type/Value
Activation Function	Selu-Selu-Relu-Relu-Elu-Selu-Sigmoid
Number of hidden layers	7
Number of units in each layer	64
Optimizer	RMSProp
Loss function	MSE
Number of Epochs of each trial	100 to 200
Train-Test Ratio	0.8–0.2

**Table 6:** Technical and software requirements.

Technical and the Software Requirements	Proprieties
Operating system OS	Windows 10
Processor	Intel Core I7 10th Gen
Programming language	Python 3.9
Platform	Tensorflow 2.3.0
Deep learning framework	Keras 2.4.3

**Table 7:** Comparison of the proposed method with the different prediction algorithms.

	Estimation Algorithm				
	LR	KNN	RFR	ANN	Proposed Taguchi Optimized Method
**MSE**	2.4 × 10^−2^	2.2 × 10^−2^	2.2 × 10^−2^	0.55 × 10^−2^	<0.2 × 10^−2^
**MAE**	1.2 × 10^−2^	10^−2^	1.6 × 10^−2^	0.6 × 10^−2^	<0.2 × 10^−2^
***R*** ^2^	0.72	0.82	0.82	0.89	0.94

## Data Availability

The data presented in this study are available on request from the corresponding author.
